# Adapting the AIS for geographic and phylogenetic research on language change dynamics

**DOI:** 10.1007/s10579-026-09956-2

**Published:** 2026-10-31

**Authors:** Borja Herce, Michele Loporcaro

**Affiliations:** University of Zurich, Zürich, Switzerland

**Keywords:** Diachrony, Dialectology, Lexicon, Phonology, Romance, Speed of change

## Abstract

Traditional linguistic atlases can be leveraged to investigate the dynamics of language diversification and change but are severely underexploited for this purpose. Here we present a cleaned up and augmented dataset derived from the AIS (Swiss-Italian Atlas) which allows us to explore the amount of innovation at different linguistic levels (lexicon vs. phonology), and the geographic, societal, and evolutionary factors predicting the amount of change in different varieties. Results point towards a significant correlation in the speed of lexical and phonological change, as well as the greater relevance of extralinguistic variables (most importantly population size) to predict lexical innovation.

## Introduction

Traditional dialectal surveys (e.g. AIS (Atlante Italo-Svizzero), ALF (*Atlas Linguistique de la France)*, ALI (Atlante Linguistico Italiano), ALPI (Atlas Lingüístico de la Península Ibérica), etc.) are often monumental works that over several years (sometimes decades) document an enormous amount of information on the linguistic varieties spoken in hundreds of geographic locations. Despite the wealth of data (phonological, morphosyntactic, and lexical) that they have to offer, and despite our increasing ability as a field to work with and process effectively large data volumes, these resources are still severely underused in contemporary quantitative linguistic research. The main reason for this is that the methods and formats of most of these surveys predate the computer era and hence are not always suited for automated manipulation. Inconsistent transcription practices, incomparable data points, and other problems hamper the computational usability of these resources even after digitalization (which is, fortunately, increasingly pursued, see García Mouton et al., 2016, Loporcaro et al., 2021). Effort has to be put, hence, on increasing the usability of these resources, and their overall visibility outside traditional dialectology and dialectometry (Séguy, 1971; Goebl, 1982; Nerbonne, 2009), as they have a great untapped potential to tackle current topics on language change and its predictors (see Wieling et al., 2011, De Vogelaer & Seiler, 2012, Wieling & Nerbonne, 2015).

In this paper, we present an upgrade to the AIS data (a clean-up and selection of variables, and the adding of Classical Latin and etymological information) aimed at improving the usability of the original data to address questions related to language change. More specifically, the added information allows us to separate innovations from retentions and to explore separately different types of change (e.g. lexical vs. phonological). Thanks to this information, we will be able to ask among other things if the amount and speed of change at different levels is correlated (see Heeringa & Hinskens, 2011, Greenhill et al., 2017), and whether extralinguistic aspects like geographic isolation or population sizes can predict the rates of change in different varieties (see Wichmann et al., 2008, Wichmann & Holman, 2009, Bromham et al., 2015).

The typological literature is teeming with hypotheses on the effect of extralinguistic variables on language structures and language change. The Linguistic Niche Hypothesis (see Dale & Lupyan, 2012), for example, argues that the social environment where a language is spoken (e.g. the size and structure of the speech community) drives language structures in a way which, like selection in biological evolution, increases their fitness within their host community. Alternatively, properties of the speech community like population size can influence change or its speed without the need of selection, for example through the different effects of drift in structurally different populations (see Reali & Griffiths, 2010). In either case, and although some disagreement persists (Scherbakova et al., 2023), there is accumulating converging evidence that larger and more highly-connected speech communities tend towards larger vocabulary and simpler grammar (Deutscher, 2010: p. 111, Reali et al., 2018). Stable social networks and low numbers of second language learners (or in other words geographic isolation and primarily ‘esoteric’ communication, see Wray & Grace, 2007) is also thought to promote morphological complexity and to also be associated with language conservatism generally (Marshall, 2004; Trudgill, 2020).

When it comes to language splits (i.e. ‘speciation’ in biology), dialect continua like Romance are also particularly exciting in relation with topics such as the interaction of phylogenetic and areal dependencies (see e.g. Koile et al., 2022), tree-like vs. wave-like diversification modes (François, 2015, Jacques & List, 2019), the difference between languages and dialects (see Wichmann, 2020; Herce, 2026a, b), and the possible effects of clade-formation on the speed of change (Atkinson et al., 2008). In this latter respect, some recent research has argued that, maybe because speakers use linguistic differences for signalling community membership during split events, the speed of change accelerates during these events. This, or alternatively the social dynamics of mutual intelligibility, could explain the alleged tendency (Wichmann, 2020, 2023) for speech varieties to be either close enough to be considered dialects of a single language, or different enough to be clearly considered different languages. The plentiful data on hundreds of close speech varieties that is documented in linguistic atlases could, if properly processed, contribute important evidence to these debates.

The structure of the rest of this paper is as follows: Sect. 2. presents the AIS data and its step-by-step selection, processing, and enrichment here to allow computational and diachronic investigation. Section 3. builds a preliminary phylogenetic tree of the AIS varieties as an aid to further investigation. Section 4. quantifies the amount of change from Latin to Romance in the different varieties at the lexical and phonological levels and finds that these are significantly correlated. Section 5. explores the impact of possible extralinguistic predicting factors like population size, contact, and isolation. Finally, Sect. 6. features an interpretation of previous findings and discusses their place in current typological research on language change dynamics, and Sect. 7. summarizes the paper and its conclusions.

## Data

As advanced in Sect. 1., one of the main points of this paper is that traditional linguistic atlases can and should get a second life in the computer age. Given the wealth of data they make available and our increased ability to handle these large volumes of data, atlases like AIS, ALF, ALI, ALPI, etc. should be used much more in current research exploring language change and diversification dynamics. Alongside the lack of awareness in some circles that these old resources even exist, the main obstacle that keeps these data underused is the fact that, because these data predate the age of computers, they are often formatted in ways that are little conducive to automation. Entries in different varieties, for example, do not always match in what they contain: one may give the singular and another one the plural form of a noun, one may show the noun alongside an article and another the noun on its own, etc. These minutiae are easily ignored (maybe even harnessed as additional information) by a trained human linguist, yet they pose a significant obstacle to the automated noise-free processing of these forms. Traditional atlases, hence, need varying degrees of largely “manual” pre-processing before they are safe to use computationally.

Here, we will focus on the AIS (Jaberg & Jud, 1928) and its digitised version (Loporcaro et al., 2021), and on the usability of this resource in computational contexts. Table 1 shows some of the inconsistencies that in the original resource hamper automated treatment. Leaving the strange and out-of-place diacritics aside that result from the OCR of the original hard copy, we can see that while a separator “;” demarcates different inflected forms of the same noun in some entries (e.g. la montˈaɲa; le mo̞ntˈaɲe), it demarcates different lexical concepts in others (e.g. lu ɡˈoːju; la s̬ˈɔːla). Furthermore, such use is inconsistent even within the same map, as sometimes no separator is given to separate the different meanings (e.g. la mʊntˈaɲɐ), or these appear in the wrong order (e.g. ɐl rˈopɐ; rupˈaː). Sometimes partial rather than whole word forms are given (e.g. la montˈaɲɐ; i -ɲe) to save space (paper in the original atlas), sometimes articles are provided but sometimes not (e.g. muntˈaɲa; -ɲe), etc.

**Table 1:** Some illustrative entries of AIS (Jaberg & Jud, 1928, Loporcaro et al., 2021). The column IPA was added by Loporcaro et al., 2021

AIS	IPA	Location	Map
la muntáña; lẹ mọntáñẹ	la muntˈaɲa; le montˈaɲe	236 – Branzi	421 – LA MONTAGNA
la mọntáñα; i -ñẹ	la montˈaɲɐ; i -ɲe	237 – Gromo	421 – LA MONTAGNA
mọntáñẹ _p_. mū́ć _p_. ^┴ ┴ ┴^	montˈaɲe _p_. mˈuːtʃ _p_. ^┴ ┴ ┴^	238 – Borno	421 – LA MONTAGNA
la muntā́ñα; i -táñ, i mų̄́nt	la muntˈaːɲɐ; i -tˈaɲ, i mˈʊːnt	243 – Canzo	421 – LA MONTAGNA
la mųntáñα	la mʊntˈaɲɐ	244 – Sant’Omobono	421 – LA MONTAGNA
muntáña; -ñẹ	muntˈaɲa; -ɲe	245 – Stabello	421 – LA MONTAGNA
il kǭ́yo; i^l t^sǭ́lo	il kˈɔːjo̞; i^l t^sˈɔːlo̞	612 – Montefiascone	1568 – IL CUOIO; LA SUOLA
: kọrā́me; la sǭ́la	: korˈaːme; la sˈɔːla	615 – Leonessa	1568 – IL CUOIO; LA SUOLA
lu gọ̄́yu; la s̩ǭ́la	lu ɡˈoːju; la s̬ˈɔːla	616 – Amatrice	1568 – IL CUOIO; LA SUOLA
lā́ sǭ̀l^ə +^; ~ ~	lˈa ːsˌɔːl^ə +^; ~ ~	618 – Castelli	1568 – IL CUOIO; LA SUOLA
la sǭ́la ┴;. li^(ə)^sǭ́lα _p_.	la sˈɔːla ┴;. li^(ə)^sˈɔːlɐ _p_.	619 – Montesilvano	1568 – IL CUOIO; LA SUOLA
ęl ráwpα; ^*^ rupā́; ^*^ il rúpα	ɛl rˈawpɐ; ^*^ rupˈa; ː^*^ il rˈupɐ	1 – Brigels-Breil	173 – RUTTARE; RUTTA 3
rupā́; rų́pα	rupˈa; ːrˈʊpɐ	3 – Pitasch	173 – RUTTARE; RUTTA 3
αl rọ́pα; rupā́	ɐl rˈopɐ; rupˈaː	5 – Ems-Domat	173 – RUTTARE; RUTTA 3
rupc̋ā́r ^+^	rupcˈaːr ^+^	7 – Ardez	173 – RUTTARE; RUTTA 3

Most linguistic atlases (and also the AIS) have volumes of data that exceed any one individual’s manual capabilities; however, the judicious computational use of these resources requires quite extensive manual preprocessing of the data to unify the information in different entries. One needs to make sure that the forms in different varieties consistently include or exclude articles, that they have the same morphosyntactic values, etc. Most uses would additionally demand supplementing the raw phonological information with others (e.g. the cognacy or lack thereof of words in different varieties), and possibly selecting the most useful variables given a particular goal. In the section below we enumerate the procedures and criteria we have used in this manual clean-up and upgrade of the AIS data.^1^

Like other linguistic atlases, the AIS was elaborated on the basis of a long questionnaire which was deployed in the Romance-speaking areas of Italy and Switzerland from 1919 to 1928.^2^ It documents a mix of grammar- and lexicon-centered questions, with a mix of basic and specialized vocabulary in the latter (special attention was placed on agriculture, animal husbandry, and the natural environment). The raw resource contains 1705 datapoints (a phonetically-transcribed response, which can be either a word, or a small phrase) across around 407 locations.^3^ Originally in paper, this resource has been digitised (OCRd) through two successive projects at the University of Zurich (Loporcaro et al., 2021) and the original orthography has been converted into an IPA transcription.^4^ Starting from the digitized version available online, and besides the mostly manual clean-up of inconsistencies explained in Table 1, the following procedures have been implemented to select an optimal subset of variables and expand their quality and usability for modern computational linguistic research.

Because distinguishing between ancestral states and innovations is crucial for the diachronic use and interpretation of the data (see Boula de Mareüil et al., 2025), all maps have been **supplemented with Classical Latin data** (added as Location/Variety 0) as an additional variety ancestral to most others (all except for the Greek and Albanian varieties named in Footnote 2). Another aspect to enhance the diachronic usability of the AIS would be to be able to separate lexical and phonological innovation, which involves discriminating between cognate and non-cognate responses. Looking for example at the word denoting ‘white’, Latin/ˈalbus/and 15 – Mathon/ˈalf/go back to the same proto-word, while/ˈalf/and 41 – Cavergno/bjˈɛːnc/go back to different ancestral words. This information has been added by **supplying a cognate set identifier** (in the form of consecutive letters: A, B, C, etc.) to every datapoint to signal which words are etymologically related (same letter) or unrelated (different letter). Besides the selection of everyday basic vocabulary (inherent, to some extent, to the AIS questionnaire), no attempt has been made to weed out borrowings from vocabulary which has been inherited without discontinuities. This is so because, given the similarity of closely-related speech varieties, it would be often impossible to separate inherited from borrowed words consistently.

Related to these data additions, various further methodological aspects and conventions must be mentioned, which were aimed at maximizing the data quality of both the Classical Latin forms, the cognate coding, and of variable selection and processing.

-When multiple inflectional forms existed in Latin for a given concept, the inflected form ancestral to the documented modern Romance reflexes has been supplied. In nouns and adjectives this is almost always the accusative singular form (without/h/or final -m since these were lost ancestrally). Nouns which have different surviving inflected forms in different Romance varieties (typically Latin nominative singular in some vs. accusative singular in others, e.g. *homo* vs. *hominem*, *presbyter* vs. *presbyterum*, *latro* vs. *latronem*, etc. but also sometimes singular vs. plural, or sometimes also alternative forms of the same word (e.g. *sanguinem* vs. *sanguem*) have been ignored, as choosing a single form as ancestral across the board would be a misrepresentation.
-Words have also been excluded where Classical Latin had alternative synonyms that have left reflexes in modern Romance (e.g. *serpens* and *colubra* for ‘snake’, *timor*,* metus*,* pavor*,* formido* for ‘fear’, *spelunca*,* caverna*,* antrum* for ‘cave’, *pregnans* and *gravida* for ‘pregnant’, *argilla* and *creta* for ‘clay’, *penna* and *pluma* for ‘feather’, *magida* and *mactra* for ‘kneading trough’, *fascis* and *fascina* for ‘faggot’, *pilus* and *capillus* for ‘hair’). In the rare cases where more than a single alternative form was documented in the Romance varieties in AIS, the last one has been uniformly chosen to avoid biases. As an additional step to achieve one single form per concept and variety, segments reported as optional were uniformly discarded.
-Also discarded were those data points where we do not know what the word for some concept was in Latin. This can be due to either the limits of the historical corpus (e.g. the words for ‘scarecrow’ or ‘dragonfly’^5^ in Latin are simply not recorded), or because the concept itself did not exist (e.g. for *cucurbita pepo*, a plant originally from the Americas). Changes in (co)lexification between ancient and modern times also need to be taken into account. In Roman times, for example, carrots and parsnips were not distinguished and the name used to denote them (*pastinaca*) did not therefore mean strictly the same as either modern vegetable (in fact we find reflexes of Lat. *pastinaca* under both modern senses).
-Concepts whose referents have an uneven geographic distribution within the Romance geography have also been avoided (e.g. cypress trees and *Pistacia Lentiscus* grow only in the south of the AIS geographical domain, while *Vaccinium vitis-idaea* and *Rumex alpinus* grow, and hence have a local word, only in the north). This exclusion is simply due to the lower coverage of locations, and hence lower usability of these entries denoting geographically restricted concepts compared to universal ones.
-Also avoided were words where onomatopoeia, sound symbolism, or nursery language influences are common (e.g. cuckoo, snore, mum, grandpa). While these might be highly interesting to address some future questions, they are likely to include high levels of noise for the ones we want to explore in this paper due to similar-sounding words which are polygenetic, that is, neither borrowed nor inherited.
-In cognate coding, derivatives have been coded as different lexical items from the base. The word for ‘comb’ at Claut,/petinˈela/, is coded as not cognate with either/pˈɛteŋ/at Cencenighe (< Latin/pˈektine/),/petinˈiːna/at Siena, or/pətːənˈe̞sːa/at Roccasicura. Whenever a response or lexical entry consists of a phrase (e.g./pjˈeto lˈɛrk/, ‘comb long’ in San Vigilio di Marebbe, or/pˈɛtːɐne ɡrˈɔsːo̞/‘comb big’ in Montefiascone), the head noun was selected/kept for annotation (i.e. pjˈeto, pˈɛtːɐne). Not coded as different cognate sets are differences in the linear ordering of elements, either at the segmental level (e.g. metathesis like/tˈɛpːene/at Fonni), or, although this occurs very rarely, at the level of larger constituents.^6^

This method and executive decisions have resulted in a final dataset with 349 concepts; all cleaned up (i.e. with OCR mistakes and the original transcription idiosyncrasies fixed), cognate-coded, and with Latin added as an additional variety representing the ancestral state. In total, 132,080 datapoints have been coded,^7^ each of them made up of the following:

-A phonologically transcribed word (e.g./tʃadˈena/). Both the original fine transcription and a derived broad phonemic transcription are provided.
-The lexical concept expressed by the word, which generally corresponds one-to-one to an AIS map (e.g. ‘chain’, map 217).
-The location/variety where that word comes from (e.g. 326 – Claut). Classical Latin has been added as Location 0 and is now the only ancient variety.
-A cognate set identifier. Cognate sets are given successive alphabetical labels (i.e. A, B, C, etc.), with the cognate set ancestral to Romance (i.e. the one observed in Latin) always indicated with ‘A’.

Some illustrative entries of the final cleaned-up and augmented resource are displayed in Table 2. This is made freely available for download as the file forms.csv in UTF-8 encoding at https://osf.io/f8t7a/?view_only=5d969b2d6a834c66ba87b4eb1fd0d279.

**Table 2:** Structure of the final cleaned-up and augmented AIS-based dataset forms.csv

Form	Cognate_set	Location	Map
frˈunt	A	289 – Bagnolo San Vito	99 – LA FRONTE
frˈɔːnt	A	290 – Godiasco	99 – LA FRONTE
frˈunt	A	305 – San Vigilio di Marebbe	99 – LA FRONTE
vˈiːs̠	H	307 – Padola (Comelico Sup.)	99 – LA FRONTE
vˈiːs̠	H	310 – Piazzola (Rabbi)	99 – LA FRONTE
vˈiːs̠	H	311 – Castelfondo	99 – LA FRONTE
tʃɛrnˈiɐli	I	318 – Forni Avoltri	99 – LA FRONTE
s̪arnˈeːli	I	327 – Forni di Sotto	99 – LA FRONTE
tʃarnˈɛːli	I	328 – Tramonti di Sotto	99 – LA FRONTE
lˈi̇ŋɡa	A	744 – San Chirico Raparo	106 – LA LINGUA
wˈi̇ŋə	A	745 – Oriolo	106 – LA LINGUA
ɡlˈɔsːa	B	748 – Corigliano d’Otranto	106 – LA LINGUA
lˈi̇ŋwa	A	749 – Salve	106 – LA LINGUA
ɟˈuːfa	C	751 – Acquaformosa	106 – LA LINGUA
lˈi̇ŋɡa	A	752 – Saracena	106 – LA LINGUA
lˈiŋɡwa	A	780 – Condoni (Briatico)	106 – LA LINGUA
ɡlˈɔsːa	B	792 – Ghorio (Roghudi)	106 – LA LINGUA
lˈi̇ŋɡwa	A	794 – Benestare	106 – LA LINGUA

In the spirit of the CLDF data format (Forkel et al., 2018),^8^ additional csv files provide further information about forms, locations, and maps:

- Symbols.csv provides information about the forms, namely the phonetic features of the segments employed in the transcription (i.e. +/- bilabial, +/- alveolar, etc.). We used Mortensen et al.’s (2016) Panphon as a source for the phonetic features of different IPA characters (see Table 3).
- Locations.csv provides information concerning the various locations in AIS: geographic latitude and longitude, population sizes, and ad hoc measures of geographic isolation and language contact to be discussed in Sect. 5. Glottocodes have also been supplied manually to match the affiliation of the different local dialectal varieties to different languages (see Table 4).
- Concepts.csv provides information about the meanings documented in the AIS maps. We rely on the concept entries of List’s et al. (2016) Concepticon whenever possible, which we supplement with a usage frequency count (see Table 5). We derive this measure of frequency from the Google Books Ngram Corpus (see also Michel et al., 2011, and Lin et al., 2012) of Italian. In order to reflect a long-term historical usage, we retrieve the 50-year rolling average in 1900.^9^

**Table 3:** Structure of symbols.csv

Ipa	Syl	Son	Cons	Cont	Lat	Nas	Strid	Voi	Ant	Cor	Distr	Lab	Hi	Lo	Back	Round	Velaric	Long
ɖː	-	-	+	-	-	-	-	+	-	+	-	-	-	-	-	-	-	+
t	-	-	+	-	-	-	-	-	+	+	-	-	-	-	-	-	-	-
d	-	-	+	-	-	-	-	+	+	+	-	-	-	-	-	-	-	-
tː	-	-	+	-	-	-	-	-	+	+	-	-	-	-	-	-	-	+
dː	-	-	+	-	-	-	-	+	+	+	-	-	-	-	-	-	-	+
p	-	-	+	-	-	-	-	-	+	-	0	+	-	-	-	-	-	-
b	-	-	+	-	-	-	-	+	+	-	0	+	-	-	-	-	-	-
pː	-	-	+	-	-	-	-	-	+	-	0	+	-	-	-	-	-	+

**Table 4:** Structure of locations.csv

Location	Country	Lat	Long	Population	Contact	Isolation	Glottocode
17 – Lenz	Switzerland	46.68	9.56	302	4	0.246	roma1326
19 – Zernez	Switzerland	46.70	10.09	760	4	0.261	roma1326
22 – Olivone	Switzerland	46.53	8.95	748	3	0.653	lomb1257

**Table 5:** Structure of concepts.csv

Map	Concepticon_ID	Name	Definition	Semantic_field	Ontological_ category	Frequency
99 – LA FRONTE	123	FOREHEAD	The part of the face between the hairline and the eyebrows.	The body	Person/Thing	0.012315
137 – IL CUORE	1223	HEART	A muscular organ that pumps blood through the body.	The body	Person/Thing	0.016421
196 – IL PORRO	NA	LEEK	A cultivar of Allium Ampeloprasum	Food and drink	Person/Thing	6.21E-05
75 – MORIRE	1494	DIE	To cease to live.	The body	Action/Process	0.00301

**Fig. 1:**
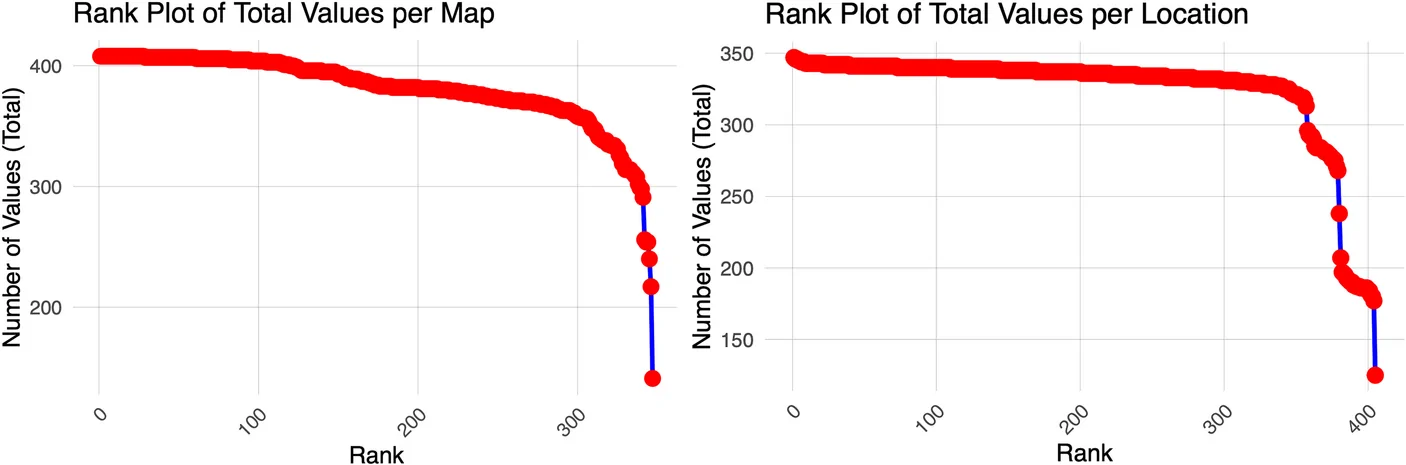
Coverage of locations (left) and concepts (right) in the final curated dataset

The final dataset is quite well saturated (i.e. ‘dense’ in the terminology of Graff et al., 2024), in that most locations have most concepts documented, and most concepts are documented for most varieties. Figure 1 shows this coverage visually. In terms of maps (left), over 300 concepts are documented for over 350 locations. In terms of locations (right), over 350 locations have over 300 documented concepts.

**Fig. 2:**
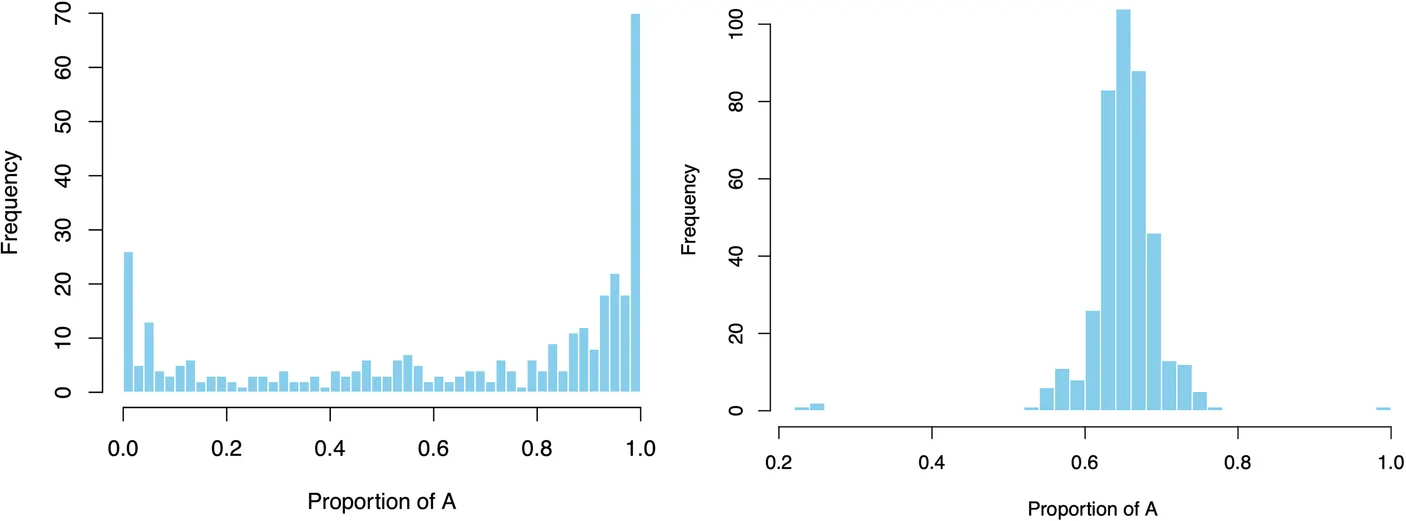
Average shared cognacy with Latin by concept (left) and location (right)

The concepts include a mix of stable and variable ones. As shown in Fig. 2 (left panel), many of the documented concepts have preserved the inherited Latin word (coded as A) in most locations. These highly stable concepts are useful to investigate phonological evolution and innovativeness in different varieties. The other less stable concepts are most useful for investigating lexical evolution and the internal subgrouping of Romance, and lexical and phonological diversification in a dialect continuum. Figure 2 (right panel) shows the degree of lexical similarity to Latin of the different varieties. The rightmost outlier is Latin itself, the language where, due to the coding convention in the present database, all concepts are tagged as cognate set A. The three leftmost outliers with the fewest cognates shared with Latin are, unsurprisingly, the three locations where non-Romance varieties are spoken (see Footnote 1). Contemporary Romance varieties in the AIS vary between 0.54 and 0.77 shared lexicon with Latin.

**Fig. 3:**
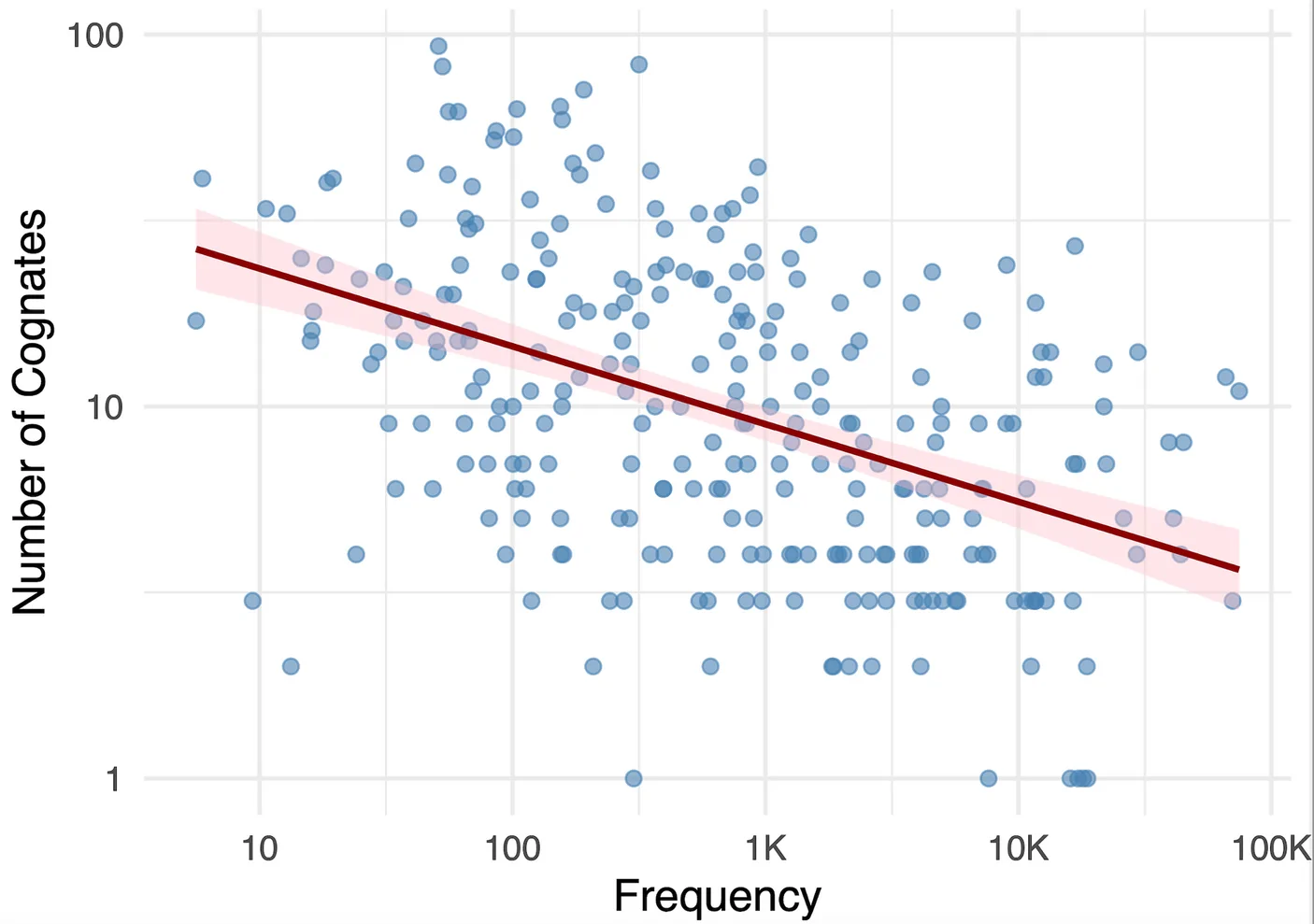
Inverse correlation of frequency and lexical replacement in our concepts

In addition to the prevalence of Latin cognates, the number of distinct cognates within a given concept is one of the main indicators of the speed of lexical replacement. While some concepts (e.g. 288 – DIECI) have a single cognate across all documented varieties, others have a very large number of etymologically different words (the top corresponding to 169 – IL MOCCIO, with 93). As might be expected (see also Pagel et al., 2007), there is a statistically highly significant (*p* < 0.0001) inverse correlation (Kendall’s Tau = −0.32) in our dataset between usage frequency and the speed of lexical replacement (see Fig. 3).

**Fig. 4:**
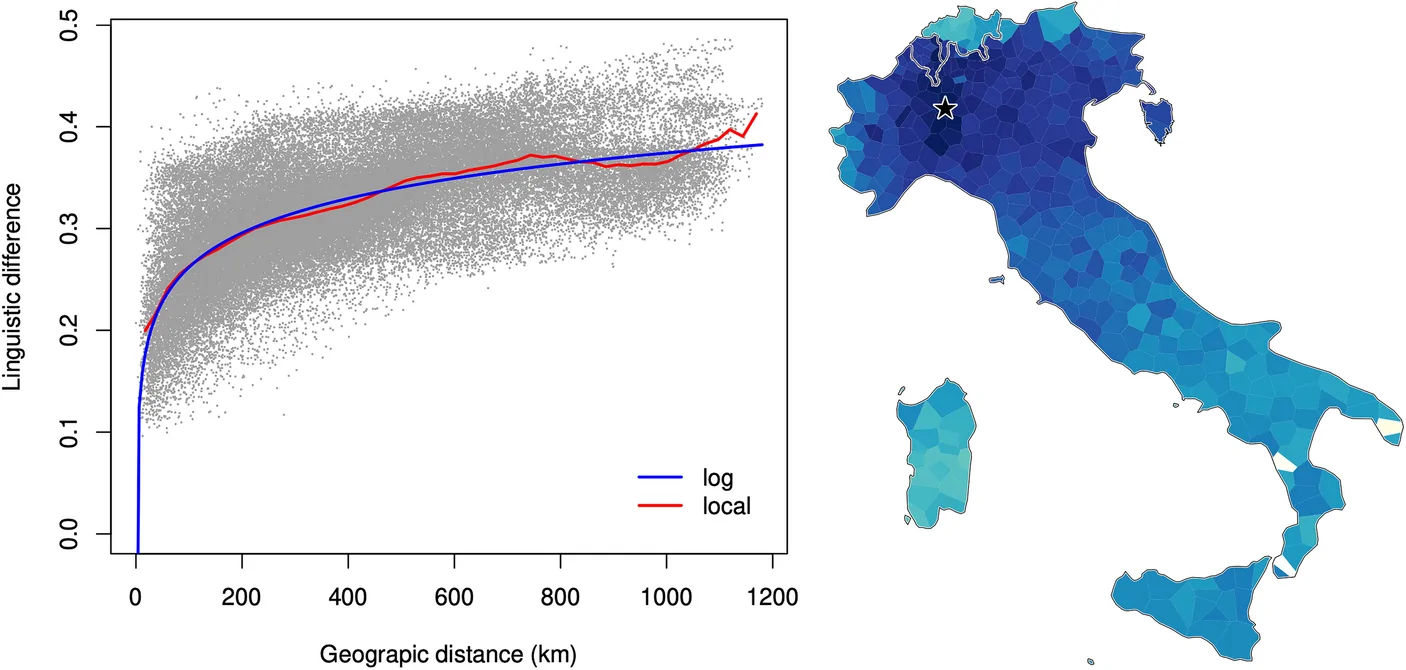
Gabmap visualizations of the correlation between word form similarity and geographic distance (left), and of the proximity regarding shared cognates (right) of all the AIS locations to point 261 Milan, with more similarity indicated through a darker shade

Thanks to existing software and applications like Gabmap (Nerbonne et al., 2011, Leinonen et al. 2016), this database (both phonetic forms and lexical cognates) can be explored, without much computational expertise, to investigate multiple aspects of Romance linguistic diversity, such as the well-known correlation between geographic proximity and linguistic similarity (see Fig. 4, left), or to produce visualizations of the similarity^10^ between the speech in different locations (e.g. in Fig. 4 right, relative to Location 261 Milan). The following Sects. 3 through 5 will outline some more specific lines of inquiry that become accessible through this database.

## Building a phylogenetic tree of the AIS varieties

Among historical linguists, there has always been debate regarding how realistic the tree model of language diversification is relative to alternatives like the wave model (François, 2015, Jacques & List, 2019). The tree model is undisputed in evolutionary biology because after (complete) separation, gene flow between species becomes extremely rare. With cultures and languages, however, there is no such limitation. Innovations can and do spread quite freely from one language to another, crossing even family-level boundaries. This is the case of lexical changes (i.e. borrowings), as well as of grammatical and phonological innovations (e.g. the uvular pronunciation of/r/, which spread in Europe across some Romance and Germanic varieties while sparing others). The result is that speech varieties can become more like each other after separation. Contemporary Galician, for example, is closer to Castilian than medieval Galician was (see Pichel et al., 2021). This is probably due to the political unity of the two territories (i.e. Galicia and Castile) and the concomitant higher degree of contact compared to the one with Portuguese. Under this scenario, one might wonder whether wave models and networks might not be of more use than trees to capture language affinities. The key, of course, and the reason why both approaches are useful, is that they capture different realities. The current increased affinity of Spanish and Galician does not erase the fact that in the medieval period Galician was much closer to Portuguese than to Spanish, and that ancestrally the phylogenetic unity with Portuguese was longer. Different phenomena, hence, call for different methods. Rather than fixate on the limitations of either approach, one should try to harness their best qualities to assess synchronic affinities and diachronic evolutionary histories, and the degree of tree-like and wave-like diversification. As a family with both dialect continuum areas and hard language boundaries, and with a very well documented direct ancestor, Romance offers a unique opportunity to lead research on the evolutionary dynamics of language change and current hot topics like schismogenesis (Atkinson et al., 2008, Kaiping & Neureiter, 2022).

When it comes to the evidence and data used to investigate the linguistic past and build these trees or webs, lexical data is usually the main source of information. This is so because, given the arbitrariness of the linguistic sign (Saussure, 1916), and the large design space of lexical forms (say the 10,000 different phonotactically possible shapes for a root in a particular language), the same concept is highly unlikely to be expressed with the same root in different languages by chance (i.e. independently). In other words, similar or identical lexical replacements are inherently unlikely to be polygenetic events. Using, for example, a word *bucca* to express ‘mouth’ is so unlikely that, when we find two languages with this form-meaning pairing, we tend to favour either borrowing or an ancestral phylogenetic unity by which the emergence of *bucca* as the form for ‘mouth’ would have happened at a point in time when the two languages were still one, and hence the unlikely event had to happen only once. Morphosyntactic and phonological innovations and traits, by comparison, are more likely to arise independently in different languages, since these are typically motivated (e.g. ease of articulation in the case of sound change) and/or involve a choice between a lower number of possible alternative states (e.g. VO or OV). While some linguistic structures (e.g. paradigmatic morphology, see Nichols, 1996, Herce & Bickel, 2025) might match the strength of evidence of lexical correspondences, the lexicon offers many more independent datapoints, which is the reason why systematic sound correspondences in the basic vocabulary still represent the gold standard to diagnose whether two languages are related.

At the same time, several complications arise when it comes to understanding shallower within-family relations. It is well known, for example, that semantic extensions (Grice, 1991) and colexifications (François, 2008) often travel through well-trodden paths involving metaphor, metonymy, etc. (Koch, 2016; Traugott, 1985, 2017). Because closely-related languages share most of their form-meaning lexical pairings, they can also come to acquire the same root for a particular concept as a result of one of these comparatively likely semantic extensions. For example, the colexification of ‘sweet’ and ‘tasty’, or ‘sweet’ and ‘good’ is found in multiple languages belonging to different language families, and although this was not a colexification pattern ancestral to Romance, the original adjective for ‘sweet’ *dulcis* has been replaced by a reflex of *bonus* (originally ‘good’) in completely independent localities in Istria (397 – Rovigno) and Abruzzo (619 – Montesilvano). This is not an isolated development: Inherited *porcus* ‘pig’ is replaced by *animal* also in disconnected locations, and inherited *lacrima* ‘tear’ is often replaced by *acqua degli occhi* (lit. ‘water from the eyes’) or similar phrases. Identical but in all evidence independent innovations, hence, are possible in the context of an inherited shared lexicon and common patterns of semantic change.

Another limitation of lexicon-based approaches is that they generally incorporate a “cheat” in the form of ‘cognacy’ and the weeding out of inherited items from borrowings. This separation of older vs. newer shared lexical patterns is only possible by bringing phonological change as well into the method. We know, for example, that English *have* cannot be cognate with Latin *habere* because Germanic/h/is expected to correspond to/k/in Latin (e.g. *horn* vs. *cornu*, *hundred* vs. *centum*, *heart* vs. *cor*). If two language varieties are compared where no separate phonological changes have occurred in a given word, it is impossible to decide if the sharing of identical roots for the same meaning is due to inheritance or due to contact. Furthermore, these might not even represent alternative scenarios ontologically: contact with the neighbouring variety might reinforce the retention of the inherited item. Given the aforementioned difficulties and the absence of fully worked out historical phonologies for the documented AIS varieties, no attempt has been made to weed out inherited items from borrowings in cognacy annotation. Given the selection of words (mostly everyday common vocabulary which should be quite resistant to borrowing) and the focus on quite closely related varieties, we believe this limitation will not affect the general validity of the data.

**Fig. 5:**
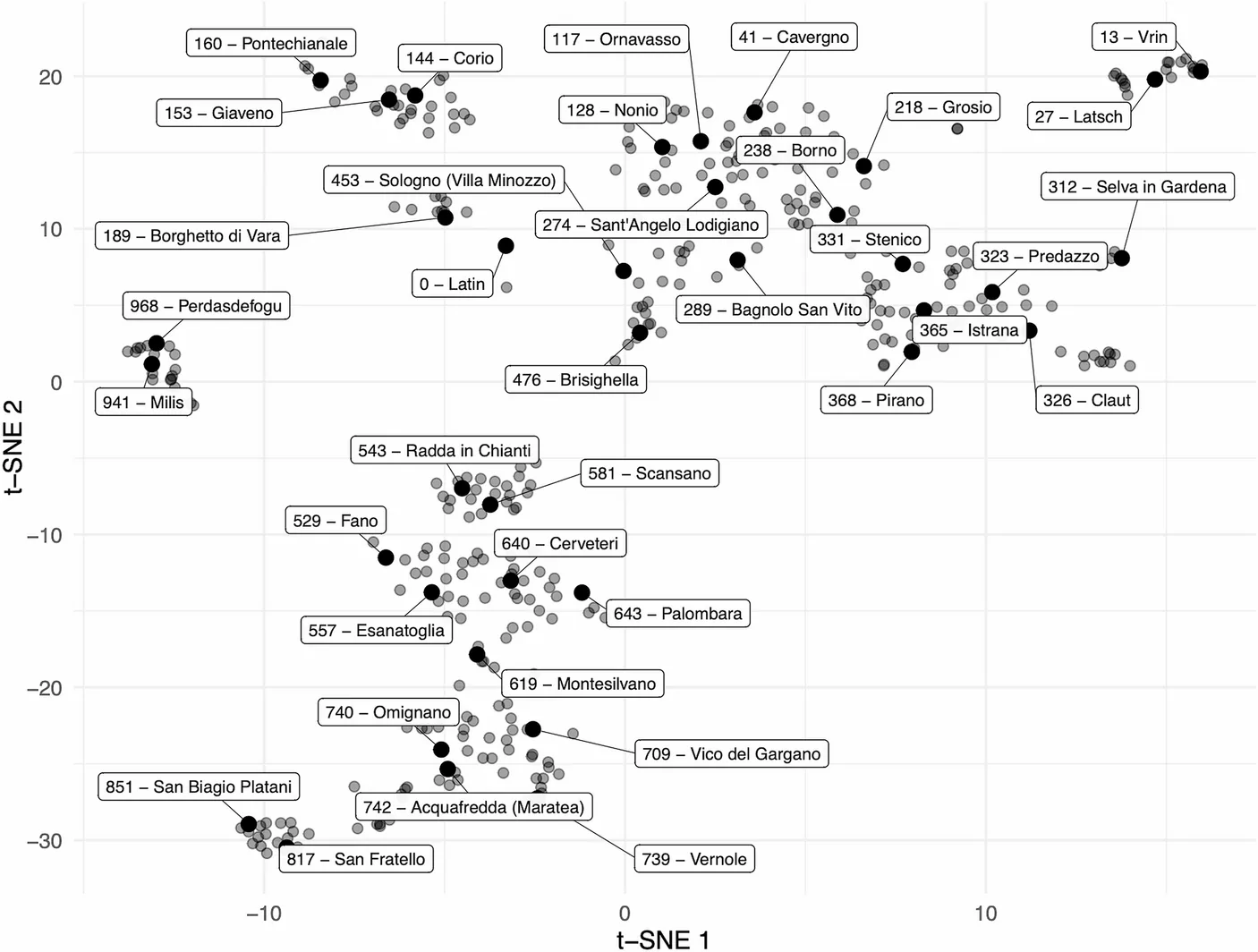
t-SNE visualization of lexical similarities among the AIS varieties

Looking at between-location lexical similarities (i.e. proportions of shared cognates), in fact, the traditional linguistic divisions (see e.g. Loporcaro, 2009) emerge quite neatly. Figure 5 shows a t-SNE visualization (van der Maaten & Hinton, 2008) of lexical variation among the AIS varieties. This is a technique similar to PCA for visualising high-dimensional data in a lower-dimensional map. It is especially useful as an exploratory tool for exploring neighbourhood structure. In the present 2D plot, each point represents one AIS variety. Points that are close together have similar (i.e. cognate) words. While t-SNE dimensions are not generally interpretable, they correspond in our case quite closely to the geographic dimensions of longitude (1) and latitude (2). We see the island of Sardinia as a tight cluster to the left/West (locations with IDs in the 900 s), the island of Sicily to the South-West (locations with IDs in the 800 s), and among mainland varieties we see a large split between varieties to the South (IDs 500–700 s) and North (rest) of the renowned La Spezia-Rimini line.

**Fig. 6:**
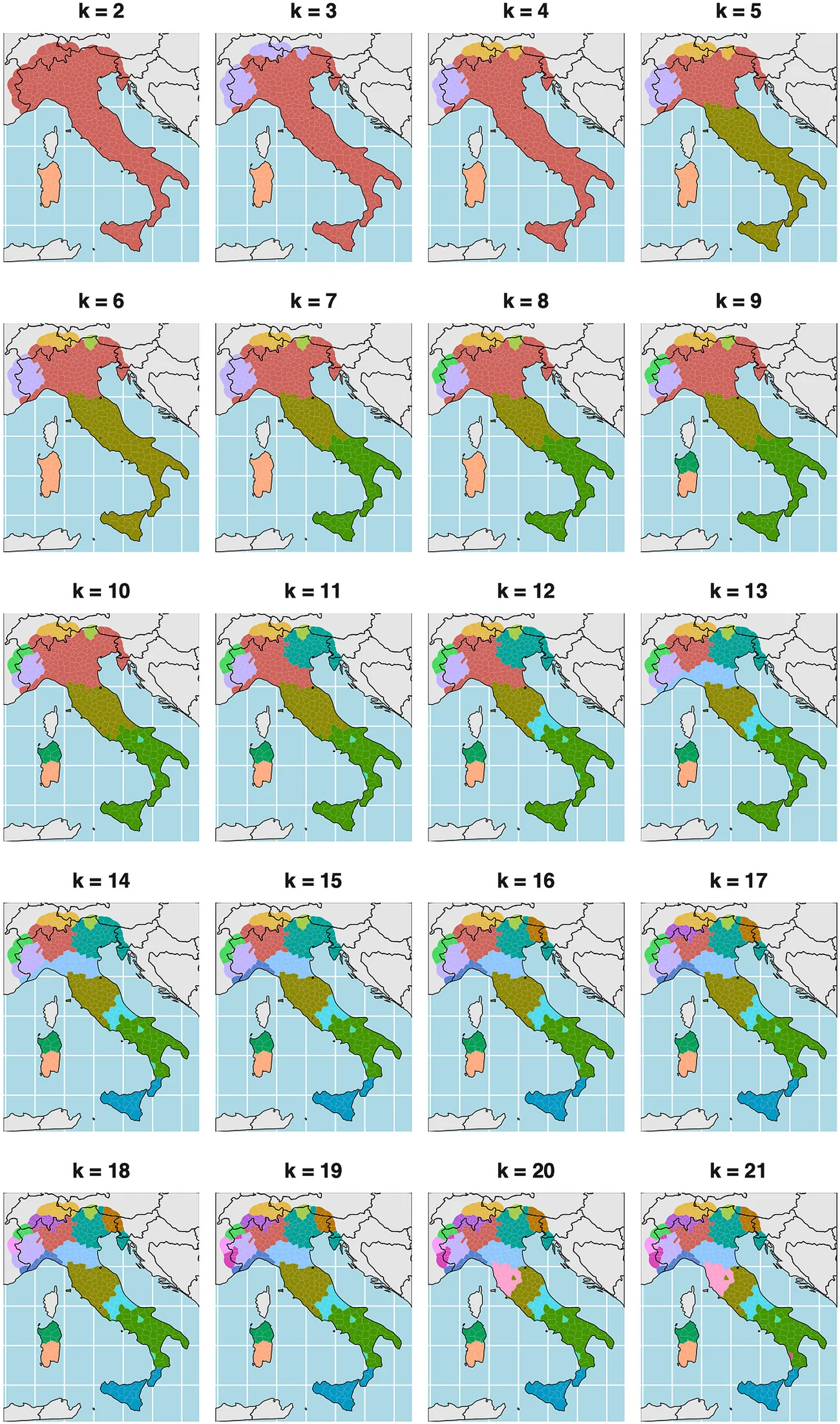
Lexical-similarity-based clustering of AIS varieties into different numbers of clusters

Laid upon the actual geographical map of Italy, Fig. 6 shows a classification of the AIS varieties into different numbers of clusters, implemented through the hclust function (method = “complete”) of the stats package (R Core Team, 2013). We see at k = 2 that the strongest lexical divide separates Sardinian from Continental Romance. At k = 5 the divide emerges between the varieties to the North (Western Romance) and to the South of La Spezia-Rimini. While the line between languages and dialects is notoriously blurry, especially in dialectal continua like these, by the time we reach 16 clusters, most of the varieties identified (e.g. by Glottolog, see Hammarström et al., 2024; Herce 2026a, 2026b) as belonging to different languages are separated. At k = 15 we get the split between Ligurian and Emiliano-Romagnolo, at k = 16 we get the split between Venetian and Friulian, at k = 18 the split between Occitan and Franco-Provençal. Some divisions often regarded as dialectal also emerge at k = 17 between Alpine and the rest of Lombard, and at k = 20 between Tuscan and the rest of Central Italian. Even the aspects that at first sight seem geographically surprising make sense upon reflection. The two separate locations split off from Southern Italian at k = 10, for example, correspond to Faeto and Guardia Piemontese, Northern Italian exclaves in the South of the peninsula that emerged from population migrations in the Middle Ages. The lexical idiosyncrasy of Romash varieties (which likely drives the very early split at k = 3 and k = 4) is very likely the result of heavy borrowing from German.

**Fig. 7:**
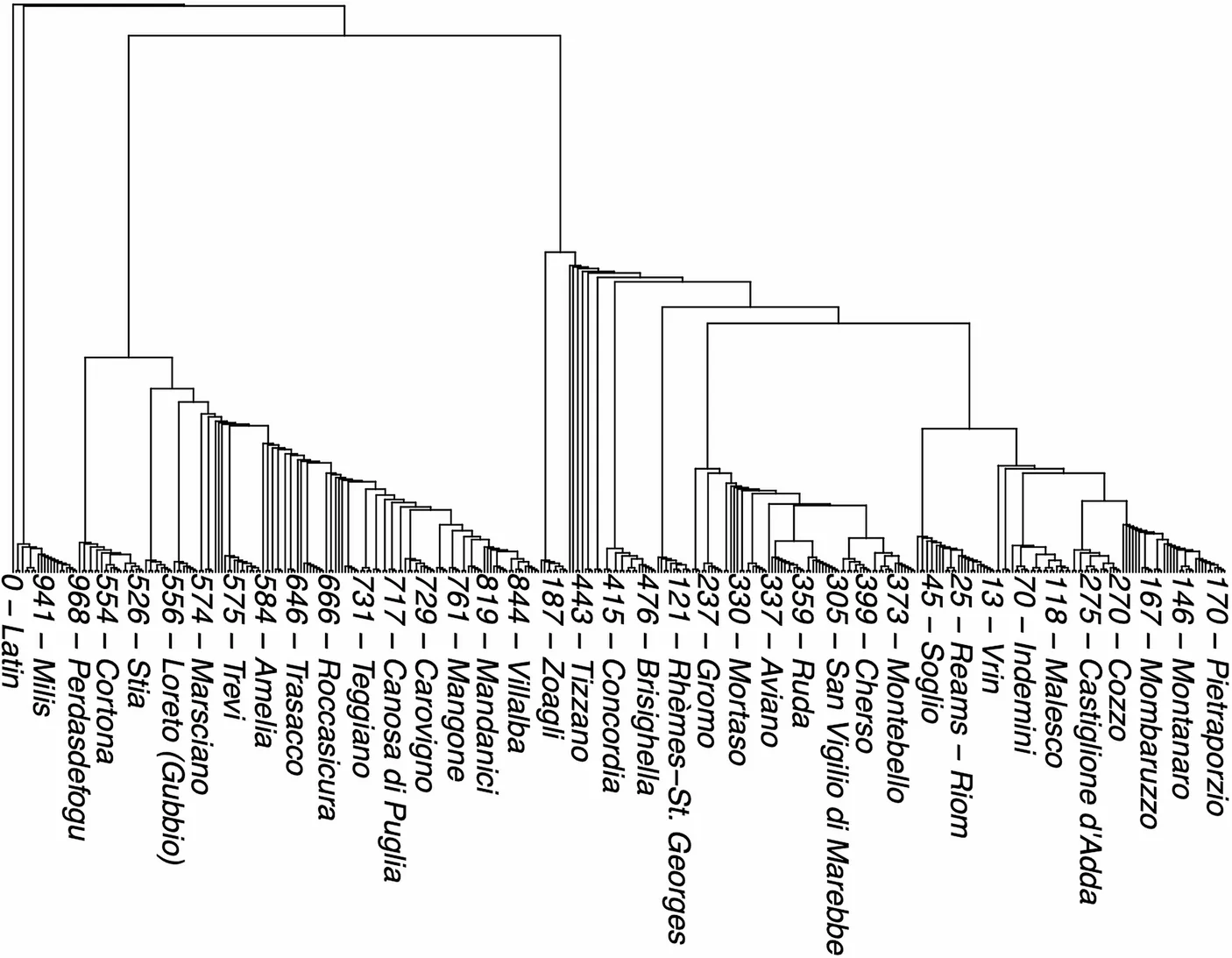
Maximum parsimony phylogenetic tree of the AIS varieties, showing only every tenth label for ease of visualization (the full tree can be inspected in the supplementary information accompanying this paper)

Because raw lexical distances can be affected heavily, as in this case, by language contact and lexical change in recent times, *historical* linguists are often (more) interested in phylogenetic trees that capture the *history* of diversification. A maximum parsimony (MP) tree (see Fig. 7) was constructed using the parsimony ratchet algorithm (Nixon, 1999) as implemented in the phangorn R package (Schliep, 2011). After excluding concepts with under 95% coverage of varieties and varieties with under 20% coverage of concepts, the analysis was conducted on a final dataset containing 365 varieties, and 3,377 cognate sets across 223 concepts. Character states were treated as unordered and equally weighted. The pratchet() function was employed with a maximum of 10 ratchet iterations, enabling stochastic branch swapping to improve tree search efficiency. The tree search included a randomized reweighting of characters across iterations to escape local optima and locate the most parsimonious tree, which had a tree length of 27,070 steps, a consistency index (CI) of 0.1757, and a retention index (RI) of 0.545 (see Kluge & Farris, 1969, Farris, 1989), suggesting moderate to high homoplasy.

**Fig. 8:**
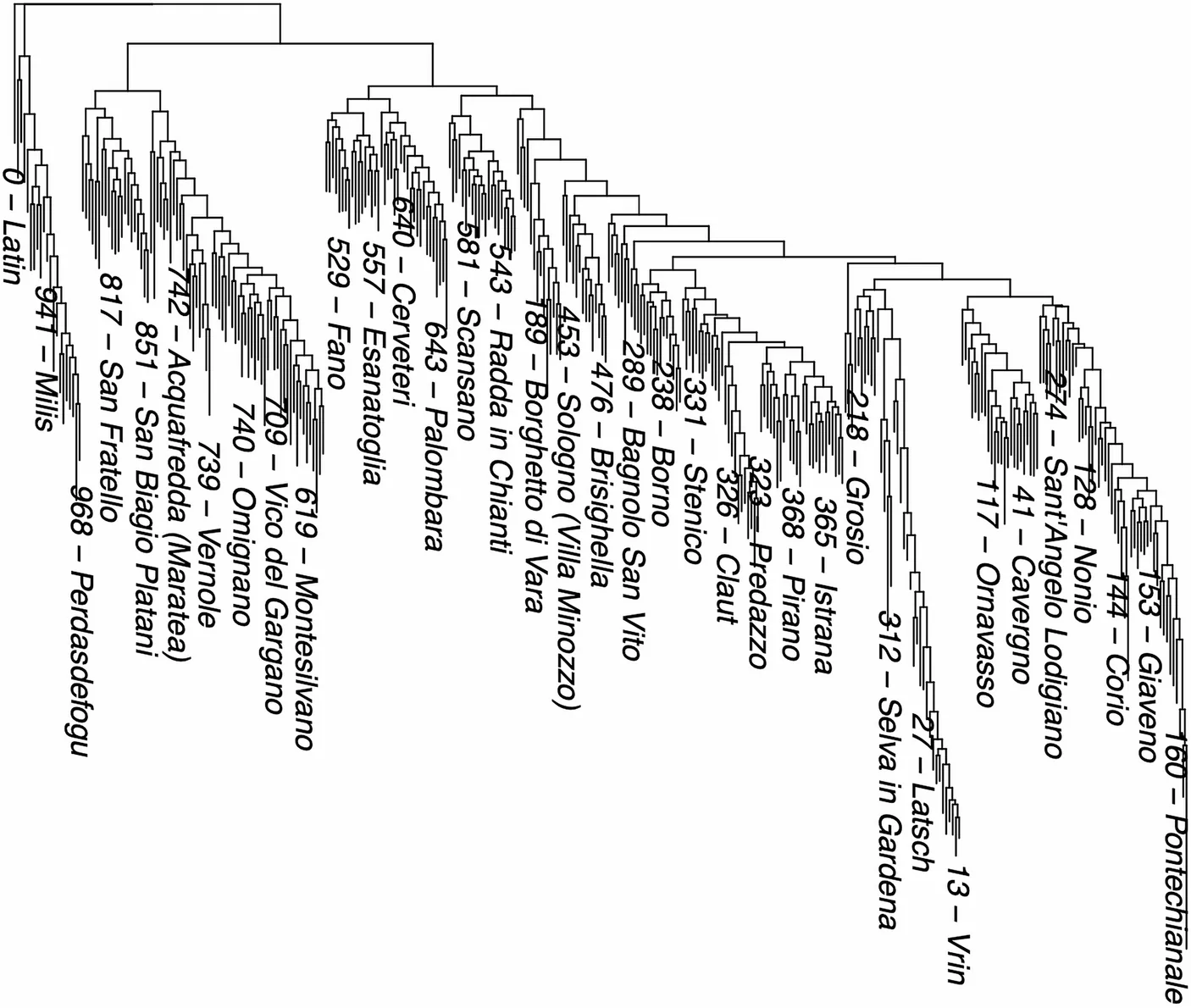
Maximum likelihood phylogenetic tree of the AIS varieties, showing only every tenth label for ease of visualization (the full tree can be inspected in the supplementary information accompanying this paper)

We also inferred (see Fig. 8) a Maximum Likelihood (ML) phylogenetic tree using the pml() function for comparison. The initial tree was estimated via neighbor-joining, and the ML tree was optimized using the optim.pml() function. Model selection followed a Markov process for discrete character evolution, using an Equal Rates (ER) model. Tree topology was optimized with Nearest Neighbor Interchange (NNI) and stochastic tree rearrangement. The optimization process involved 119 ratchet iterations, each performing topology perturbations and likelihood optimization to avoid local optima.

Despite the high degree of homoplasy (i.e. non-tree-like signal), probably expected from what is to a large extent a dialect continuum (also known more recently as a ‘linkage’, see François, 2015), the obtained tree matched the core insights of extant philological research on the split and subgrouping of Romance. Thus, the first split we found corresponds to that between Insular (i.e. Sardinian, the varieties with IDs in the 900 s) and Continental Romance (the rest), while the second one corresponds to the split between Italo-Dalmatian (the varieties of the Italian Peninsula and Sicily, with IDs from the 500 s to 800 s) and Western Romance (the rest, with IDs 0 to 400 s).

The search for an optimal tree is NP-hard, and given the size of the current dataset, with hundreds of characters and taxa, more sophisticated methodologies (Bayesian) that permit absolute dating and allow to derive the degree of confidence in different nodes would take quite long, which will be therefore left for future research. Here the main purpose is to approximate the phylogenetic structure of the varieties spoken at the AIS locations so this can be taken into account in the rest of this paper. Hence, from the ML tree, we obtain a phylogenetic variance-covariance matrix with which to control for shared ancestry.^11^ This way we also enhance further the usability of the AIS data for testing diachronic hypotheses and conducting further evolutionary research on the AIS data, as illustrated in the following Sects. 4 and 5.

## Comparing the speed of lexical and phonological evolution

Thanks to the inclusion of Latin data, one of the aspects that immediately becomes accessible is the amount of change in different varieties. Excluding the three locations where non-Romance varieties are spoken, all the remaining ones are directly descended (Goldstein, 2024) from Classical Latin. Hence, lexical and phonological discrepancies between a Latin and modern word must be interpreted as the result of change during the last 2000 years. In addition, through cognacy annotation, lexical changes and phonological changes can be explored separately.

When it comes to the lexicon, we can see if some variety, for a particular meaning, has preserved the lexical item from Latin or has instead replaced it by a different one. As the cognate set labelling in Table 6 shows, for example, locations 1 and 53 have preserved the Latin lexeme for ‘snow’ (Map 378 from AIS) while location 93 has replaced it by a different unrelated item/fjˈɔka/(ultimately from Latin *floccus* ‘tuft’). If we want to estimate the degree of lexical conservatism of a variety, thus, we simply need to calculate the proportion of A-tagged words across all the attested meanings in our dataset.

**Table 6:** Illustrative cognacy annotation for the concept ‘snow’

Form	Cognate_set	Location	Map
nˈiwe	A	0 - Latin	378 – LA NEVE …
nˈɔjv̥	A	1 – Brigels-Breil	378 – LA NEVE …
nˈøw	A	53 – Prosito (Lodrino)	378 – LA NEVE …
fjˈɔka	B	93 – Ligornetto	378 – LA NEVE …

When it comes to phonological evolution, comparing the ancestral Latin (i.e./nˈiwe/) and modern forms tagged with the same cognate set label A (i.e./nˈɔjv̥/and/nˈøw/) allows us to gauge the amount of historical phonological change. A broad phonemic transcription was adopted to avoid researcher-specific preferences in transcription and the use of unnecessary or inconsistent diacritics (e.g./v̥/in Table 3 was converted to/f/). In the simplest case,^12^ we can count the number of segments that are the same as in Latin (i.e. 1,/n/, in/nˈɔjv̥/vs. 2,/n/and/w/in/nˈøw/) and we can express this as a proportion (i.e. 1/4 = 0.25 and 2/4 = 0.5). If we want to estimate the degree of overall phonological conservatism of a variety, we simply need to calculate the average across all meanings tagged as A in our dataset.

**Fig. 9:**
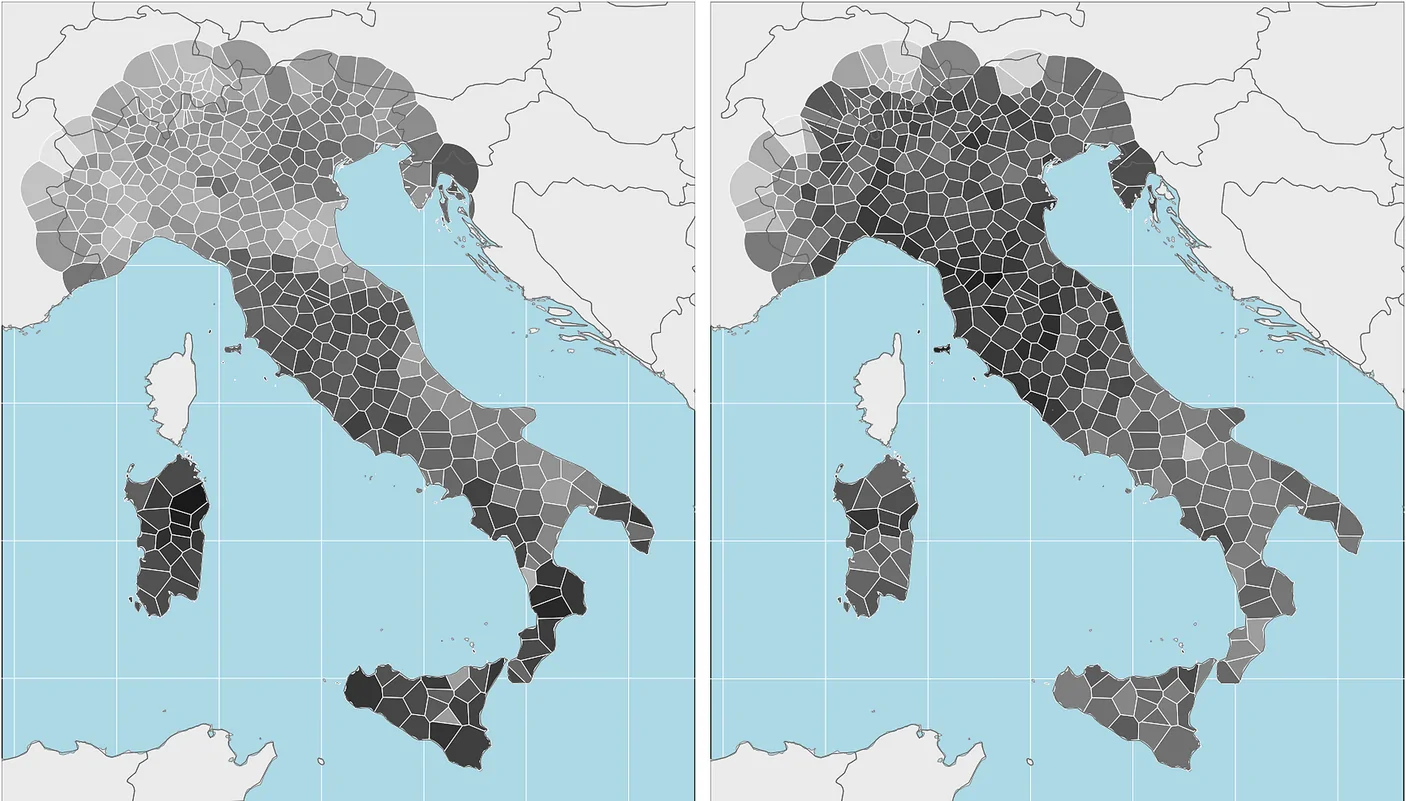
Phonological (left) and lexical (right) conservativeness (darker shade of grey) and innovativeness (lighter shade) of the AIS varieties

Figure 9 shows the degree of phonological and lexical conservatism (black) of the AIS varieties with enough data.^13^ Phonological conservatism reaches its highest value in 938 – Bitti, in Sardinia, with 0.823 (i.e. over 80% preserved segments), and by regions is highest in Northern Sardinia, Sicily, and Calabria. Phonological innovation is highest at point 121 – Rhèmes, in the Aosta valley, with 0.423, and by geographical area is most pronounced in the Alps and in Gallo-Italic: Lombardia, Piemonte, Liguria and Emilia-Romagna. Lexical conservatism, in turn, reaches its maximum value in 522 – Vinci, Tuscany, with 0.713 (i.e. over 70% preserved lexemes), and by region is highest in Central Italy, Liguria, and in North-Central Sardinia. Lexical innovation is highest at 122 – Saint-Marcel, again in the Aosta valley, with 0.531, and by regions it is most pronounced in the Alps.

These findings constitute quantitative confirmation of traditional, mostly qualitative and impressionistically deduced trends (but see Pei, 1949 for an early attempt at quantification), such as the phonological conservativeness of Sardinian, particularly of Nuorese in North-Eastern Sardinia. Language contact and isolation have been recurrently appealed to in the literature on language change, where more contact is generally associated with more change (see Trudgill, 1989, 2020, Baechler & Seiler, 2016). The present findings appear to support this broad picture. Sardinia, with its insular nature and mountainous interior, appears to have acted as a relic area not only when it comes to language here, but also regarding population genetics (Caramelli et al., 2007). Conversely, the greater innovativeness observed in the Central and Eastern Alps, particularly when it comes to the lexicon, could be associated to contact with Germanic (in Romansh in particular we observe frequent replacement of the inherited lexicon by Germanic words, such as when/wˈentus/‘wind’ is replaced by/lˈʊft/or when/pˈektus/‘chest’ is replaced by/brˈuʃt/). More detailed analysis of the predictors of phonological and lexical innovation will be conducted in Sect. 5.

Contrasting for now lexical and phonological evolution, we find that the two are significantly correlated. To examine the relationship between lexical and phonological innovativeness, we performed a Phylogenetic Generalized Least Squares (PGLS) regression. The non- independence of linguistic varieties derived from shared ancestry was incorporated into the model through a variance-covariance matrix derived from the Maximum Likelihood phylogenetic tree in Sect. 3. In this model, the proportion of preserved segments (i.e. phonological innovation) was added as an independent variable, and the proportion of preserved lexemes (i.e. lexical innovation) was included as the dependent variable. This was operationalized as a binomial response, with weights derived from the number of data points per variety, ensuring that the proportion was appropriately analyzed in the context of its underlying successes/trials structure. In this model, phonological change turned out to be a highly significant predictor of lexical innovativeness (β = 0.483, *p* < 0.001, t = 5.65), indicating that linguistic varieties with greater phonological conservatism tend to exhibit also significantly higher lexical conservatism.

It is possible (but not necessary, as will be explained in Sect. 6) that this result is due to social and geographical factors like language contact, geographic isolation, population sizes, etc. operating in similar ways on different levels of a language, in this case lexicon vs. phonology. Section 5 will be devoted to testing the effect of some of these oft-mentioned predictors of language change.

## Predicting the speed of language change in different varieties

The literature documents numerous hypotheses regarding possible extralinguistic predictors of language change. One of the most widespread proposals has involved the distinction between so-called ‘exoteric’ and ‘esoteric’ communities (Wray & Grace, 2007, Lupyan & Dale, 2010, Trudgill, 2011). It has been generally argued (but see Shcherbakova et al., 2023), that languages spoken in exoteric communities (e.g. English and other large national lingua francas) tend to be grammatically simple but lexically complex, while languages from esoteric communities (e.g. Yeli Dnye, see Levinson, 2022, and similar small local isolated languages) tend to have the opposite characteristics. When it comes to concrete factors fostering change, this dichotomy involves several logically orthogonal dimensions:

**-Population size.** For both change in languages and in population genetics, it is expected that, in smaller populations, innovations are more likely to successfully spread to the whole population (Nettle, 1999; Reali & Griffiths, 2010). This predicts that the languages spoken by smaller communities will tend to change faster, and also tend to accumulate more uncommon and even maladaptive grammatical traits due to the greater strength of drift (i.e. “random” change) relative to selection (see Raviv et al., 2019, Bromham, 2025).
**-Density of social networks.** Alongside a small population, another aspect favouring the success of an innovation is the density of social networks (see Raviv et al., 2020, see also Steiner et al., 2023). That is, in two communities of the same size, an innovation will tend to spread faster in the one that is more densely interconnected, that is, where people interact regularly on average with a greater number of other people.
**-Geographic isolation.** The possibilities for geographic mobility in and out of a particular community and area corresponds to the density of connectivity of a linguistic community with the outside. In general, it is believed that areas that are easy to reach and move across (i.e. geographically central, flat, unforested) tend to correspond to spread zones (Andersen, 1988; Nichols, 2003) where change is faster through the easier spread of linguistic innovations, and also cultures and genes. Accretion zones, by contrast, are those which are difficult to get to and move through due to physical obstacles like mountains and bodies of water.
**-Language contact.** Contact with a different language (e.g. through widespread bilingualism or a large influx of L2 learners) can lead to the adoption of foreign words, structures, and pronunciations, particularly when contact is with a socially prestigious language. In general, contact between more dissimilar languages generates more synchronic variation, which is the pool which language change draws from. Thus, more contact should be expected to speed up the pace of change (Trudgill, 1989, 2020).
**-Language splits.** Beyond the esoteric/exoteric (macro)dimension, it has been observed, in both evolutionary biology and in linguistics (Gould & Eldredge, 1977; Atkinson et al., 2008), that more change tends to happen along the branches where more language splits have occurred. The reason for this punctuated evolution in language is sometimes taken to be ‘schismogenesis’ (Bateson, 1935), which is generally defined as “the exaggeration of language differences to mark social boundaries.” (Kaiping & Neureiter, 2022).

A way can be found to operationalize and measure most of the aforementioned variables in the AIS varieties: **Population sizes** of the respective locations (at the time that the data was collected around 100 years ago) were provided in the original resource along with location names. **Geographic isolation** was operationalized through the average between two separate metrics: one capturing the roughness of the immediately surrounding terrain, and another the general peripherality of a location relative to the continental landmass. Terrain roughness (specifically the standard deviation of altitudinal differences within a 10 km-radius circle around a location) was obtained through the R packages sf (Pebesma & Bivand, 2023), terra (Hijmans, 2025) and elevatr (Hollister et al., 2025). Peripherality was approximated through the proportion of sea area within a 200 km-radius and calculated through the package marmap (Pante & Simon-Bouhet, 2013). Given the difficulty of automated calculation, a measure of **language contact** was manually provided on a 5-level scale, from 4 (most contact) to 0.^14^ The number of **phylogenetic splits** in the path to a particular variety was calculated through the package ape (Paradis & Schliep, 2019) on the basis of the Maximum Likelihood tree from Sect. 3.. No data could be reasonably obtained on the density of social networks in the different communities.

The aforementioned variables were included as predictors (i.e. fixed effects) of phonological and lexical innovation in separate phylogenetic generalized least squares models, fit through the function gls() from the nlme package (Pinheiro et al., 2025), with the phylogenetic dependence between varieties added through a variance-covariance matrix. Predictor variables are not strongly correlated (none of them show a correlation coefficient of over 0.3 or under − 0.3), which decreases the risk of multicollinearity. When it comes to predicting phonological innovation as measured by the proportion of phonemes preserved from Latin, the results in Table 7 were obtained, with a statistically significant conservative effect of isolation and no statistically significant trends in other variables.

**Table 7:** Results of the model Preserved_segments ~ Isolation + Population + Splits + Contact

	Value	Std.Error	t-value	*p*-value
Isolation	0.0528713	0.01603312	3.297629	0.0011 **
Population	0	0.00000004	−0.624127	0.5330
Splits	−0.0006989	0.00076323	−0.915676	0.3605
Contact	−0.0054368	0.00428478	−1.268853	0.2054

When it comes to predicting lexical innovation, as measured by the proportion of lexemes preserved from Latin, the results in Table 8 were obtained, with a statistically significant conservative effect of isolation once again (albeit with a smaller effect), and a statistically highly significant conservative effect of population size (i.e. larger communities tend to preserve more lexemes), something which has also been noted in recent research on the topic (Bromham et al., 2015). A highly significant effect was also found for more language splits to be associated with more lexical innovation. This (or more specifically an acceleration of change around splitting events) goes by the name of ‘punctuated evolution’ or ‘punctuated equilibrium’ in evolutionary biology (Eldredge & Gould, 1972), and has been recently argued to apply to language as well (Atkinson et al., 2008, Kaiping & Neureiter, 2022).

**Table 8:** Results of the model Preserved_lexemes ~ Isolation + Population + Splits + Contact

	Value	Std.Error	t-value	*p*-value
Isolation	0.0172724	0.006974867	2.47637	0.0138 *
Population	0.0000001	0.000000020	2.59916	0.0097 **
Splits	−0.0013858	0.000329648	−4.20378	0.0000 ***
Contact	−0.0022710	0.001858342	−1.22207	0.2225

## Discussion

This paper presents an adaptation of the AIS data (Jaberg & Jud, 1928, Loporcaro et al., 2021) that allows us to tackle evolutionary linguistics questions. It presents a cleaned-up and data augmented (Latin + cognacy information) selection of variables representing around one quarter of the original AIS dataset. One of the main goals of this paper has been to argue for the potential of traditional linguistic atlases and dialectological datasets for modern quantitative investigations of language change and diversification dynamics. While adapting these old resources to current computational methods is a painstaking endeavour, we believe it is worth it and should be pursued more decidedly and in a more coordinated fashion in the future. This enhanced subset of the AIS data is made freely available online for further research alongside the location-level data (i.e. isolation, population) that was discussed in Sect. 5. A Newick and Nexus-format phylogenetic tree of the AIS varieties is also provided to further enhance the usability of the dataset for diachronic research. We also provide the.txt and.kml files required to explore this dataset in Gabmap.

Alongside a presentation in Sect. 2 of the resource itself and the steps and method to generate it, we have also tackled, in Sects. 4 and 5 diachronic questions concerning the speed of change (or in other words the amount of change since Latin) in two different aspects of language: the lexicon and the phonology. In Sect. 4, we showed that innovation at different levels is significantly correlated. This is in line with similar previous findings in other language families (see Heeringa & Hinskens, 2011, Scherrer & Stoeckle, 2016, Heeringa et al., 2023). When it comes to our concrete linguistic levels (i.e. lexicon vs. phonology), this has two possible and mutually compatible explanations. The first is that lexical and phonological change are causally linked to each other in some way. While more complex methods, and possibly fully diachronic data, would be needed to test this (see e.g. Moscoso del Prado, 2014), it is not implausible that phonological innovation causally leads to more lexical replacement. Because regular sound change is a force that destructs morphological oppositions (i.e. Latin/kˈentu/> French/sɑ̃/, and also Latin/sˈanɡʷe/>/French/sɑ̃/, see also Herce & Marr 2026, p. 141), more sound changes may generate more proclivity for lexical replacements that can keep accidental homophony and the ensuing difficulty of comprehension and processing in check. The other compatible possibility is that other factors (e.g. sociolinguistic ones, like population sizes, the density of social networks, multilingualism, etc.) are causally associated with more/less innovation in language generally (i.e. across levels).

To explore this latter possibility, we need to understand the effect of extralinguistic factors on phonological and lexical innovation separately. This was the purpose of Sect. 5. The picture is, as is usually the case in phenomena with multiple intertwined predictors, a complex one. Our results suggest that geographic isolation could indeed have a conservative effect upon both phonology and lexicon (albeit possibly a stronger effect upon phonology). The other extralinguistic forces we investigated, by contrast, appear to have a significant effect on the lexicon only. Highly prominent in our dataset seems to be the role of population sizes. A lower population appears to be associated with an acceleration of the pace of language change. This is possibly due to drift and the greater likelihood that an emerging idiolectal feature (e.g. a semantic extension of the word for ‘silver’ to mean also ‘money’) can spread to the whole population (Nettle, 1999, cf. Wichmann & Holman, 2009). Language splitting was also found to impact lexical change, with more branching very significantly associated with greater lexical innovation. The phenomenon of schismogenesis (see Atkinson et al., 2008, Kaiping & Neureiter, 2022) injects causation into this correlation by arguing that it stems from the social use of language to signal linguistic allegiances. When two populations are diverging, the reasoning goes, linguistic differences can be exacerbated to mark these emerging social boundaries more robustly. According to our findings, lexical items appear to be more accessible to this type of modulation of linguistic traits.

The failure to find a significant impact of language contact upon both lexical and phonological innovation in our dataset is certainly surprising. Contact between dissimilar speech varieties should be expected to speed up change (for Romance see Gaeta & Seiler, 2021, Gardani, 2022), by increasing the synchronic pool of acceptable lexical and phonological variants on which diachronic change relies. Since our measure for language contact was the only one arrived at impressionistically and “by hand”, it might be simply a matter of data quality (or insufficient data) when it comes to this variable. Future research could attempt to leverage data on language use and multilingualism at different locations, but this data will most certainly be difficult to square up with the AIS locations and difficult to extrapolate to the state of language contact when the AIS data was collected.

Overall, we found that, across both types of change we explored, lexical innovation was the one with more significant extralinguistic population-level predictors. This suggests that different aspects of languages might be differentially sensitive to social pressures. The lexicon might be more prone to semi-conscious modulation (e.g. through schismogenesis) compared to phonological patterns and sound changes, which operate primarily (and regularly) due to universal articulatory dynamics dictated by the speech organs. While abundant research has been done concerning for example the borrowability of different structural categories of language (e.g. Matras, 2007 argues that nouns are more easily borrowed than verbs, and consonants more easily borrowed than vowels), more research is needed to compare the ease of borrowing in different linguistic levels (i.e. lexicon vs. morphosyntax vs. phonology, see Heeringa & Hinskens, 2014, Greenhill et al., 2017). Because of their greater tractability, external sources of change like borrowing are also researched more and are hence better known compared to internal causes of change. This paper has explored the possible role of some such predictors like the size and outward-connectivity of speech communities on linguistic diversification. It therefore represents an innovative contribution in this direction. One that uses a micro-diachronic approach and a rich but neglected dataset when it comes to understanding the predictors of language change.

## Conclusions

Profiting from the more stable and longer-term approach to science that was prevalent in the past, traditional dialectological surveys are monumental data collection enterprises with data quantities that are unparalleled by most contemporary databases. For comparison, the AIS contains approximately twice as many datapoints as Grambank (see Skirgård et al., 2023), and these individual datapoints are way richer (i.e. not dichotomous or categorical). Other dialectological surveys are much larger. Despite our greater capacity as a field to extract meaningful information from large datasets, these resources are extremely underused outside traditional philological and dialectological studies (for dialectometric explorations on the AIS see Goebl, 1981, 2008). While adapting these old resources to contemporary computational requirements requires significant work, the cost of doing so is orders of magnitude lower than the cost of generating comparable new datasets from scratch. Furthermore, the linguistic variation that some of these databases document has simply disappeared, as regional unstandardized varieties have given way to national standards in many places. It is our responsibility, therefore, to extract all “juice” out of these old materials.

The field of evolutionary language science (Bickel et al., 2024) seeks to uncover which processes, mechanisms, and change dynamics are responsible for the linguistic diversity and universals that we encounter synchronically around the world. While the breadth of data is important (because grammatical and areal similarity might generate change dynamics that are family-specific and not representative of cross-linguistic trends), we believe depth and quality of data is also paramount. Dialectological surveys afford a spatial granularity that is unparalleled. Romance, furthermore, offers an arguably unique opportunity to study language change dynamics thanks to the availability of a well-documented shared common ancestor to the family, Latin, and the ability to know rather than guess or reconstruct ancestral states. These qualities should make Romance linguistic atlases one of the hottest resources for quantitative research on language change. Romance philologists and computational linguists should work together to adapt these resources to modern formats and questions.

Our contribution in this direction has been to sketch how this could be done, cleaning up the data present in the original source (Jaberg & Jud, 1928) and its digitized version (Loporcaro et al., 2021), and adding Latin and etymological cognacy information. Thanks to these, a phylogenetic tree of the represented varieties can be put together, and the data can be used more easily to explore geographic variation and change. The main findings of this initial exploration are the validity of traditional qualitative linguistic classifications (for a summary see Cugno, 2023), and the observation that the speed of lexical and phonological change is significantly correlated. Concerning extralinguistic predictors of the speed of change, a general conservative effect of isolation was confirmed, while factors like population size and the amount of branching appeared to have an effect over the speed of lexical change only.

This resource opens up the door for quantitative research on multiple other aspects. Future work could be aimed at testing the validity of the present results with more sensitive approaches (e.g. applying more computationally demanding Bayesian methods for tree building and for tracking the evolution of traits along the tree). In addition, this rich dataset provides the opportunity to address multiple other questions regarding language diversification. One example concerns the recent claim (Wichmann, 2020, Guzmán-Naranjo & Wichmann, 2023) that linguistic distances tend to be bimodal so that two varieties will tend to be either close enough to be considered dialects of the same language or different enough to be considered different languages, with fewer varieties in-between. A dataset like the present one would allow us to check the validity of these claims with a dense web of datapoints selected for their *geographic* rather than linguistic coverage (see Herce 2026a, 2026b). In addition, it would allow us to assess if this distribution is driven by lexical replacement or phonological change, and if it emerges through punctuated evolution or schismogenesis along the family tree. We will address these and other questions in future research.

## Data Availability

This is made freely available for download as an OSF repo (link in the manuscript).

## Footnotes

1 Because the early-20th century Romance dialectal atlases that we have mentioned (AIS, ALF, ALI, ALPI) are structurally quite similar (locality grid, questionnaire, onsite fieldwork, phonetic transcription, concept-by-concept maps), the procedure outlined in this paper will be applicable in a very similar way to other atlases. A significant obstacle (which modern technologies like AI are making less and less significant every day) is the digitalization of large amounts of paper-based materials. After this step, not yet available for most of these atlases, data cleaning and annotation can be essentially the same as what will be presented here. In short: (i) extract semantically equivalent words, (ii) add Latin, (iii) add cognate coding.

2 Going into more detail, the AIS includes Romance-speaking villages in areas that used to belong to Italy until WW2 (in modern-day Slovenia and Croatia). Italy data includes answers in non-Romance enclaves in the Romance-speaking territory (more concretely responses in two Greek-speaking villages (748 – Corigliano d’Otranto and 792 – Ghorio (Roghudi) and one Albanian speaking one (751 – Acquaformosa), but does not include German-speaking villages in South Tyrol and other parts of the Alps. When it comes to Switzerland, the AIS includes data on Romance-speaking areas in the South (Ticino) and East (Grisons) but not on the Franco-Provencal and French speaking areas in the West.

3 While the numbers/IDs of locations range from 1 to 990, this is the number of locations for which data was collected. It is a network of locations which covers quite homogeneously the territory described in Footnote 1, albeit with decreasing density of sampling towards the South.

4 Details on the projects, limitations of the IPA conversion, and the downloadable data itself can be found online at https://www.ais-reloaded.uzh.ch/.

5 The word *libellula* was coined by Carl Linnaeus in the eighteenth century.

6 The name for *Mustela Vulgaris* in Sardinia offers a curious case where the subparts of a morphologically complex response are cognate but appear in a different order. The animal is named, in a seemingly arbitrary way, after the words for ‘mouth’ (buk: a, uk: a) and ‘honey’ (mɛ:li, mɛ:le). These can lexicalize the concept either as a phrase (e.g. ‘bukːa e mˈɛːle’) or as a compound with either order (e.g./bukːamˈɛːli/,/melimbˈukːa/). All these variants have been classified as cognate.

7 This number does not reach 349*407 = 142,043 due to missing responses in some locations, to be shown in Fig. 1.

8 A fully CLDF-compliant dataset is also provided online. This makes it possible to use various tools that have been developed to work with such datasets in a computational-diachronic framework, conducting among others multiple sequence alignment, or automating the inference of sound correspondences or cognates (List, 2014, 2019).

9 We are aware that usage frequencies vary between languages, genres, and across time, but there is insufficient data available for most varieties and historical periods. Because word frequencies in different languages are strongly correlated (see Calude & Pagel, 2011), we believe Italian frequencies at this point in time will constitute a good proxy for the average usage frequency of the concepts across time and space.

10 In this case, what is shown is ‘zero-based quadratic distances’. For each site, Gabmap first calculates linguistic distance from the chosen reference site as the average edit distance across all compared words. These are then rescaled from a true zero point, where zero means identical to the reference site, and squared to give the zero-based quadratic distance used for map colouring.

11 A phylogenetic variance-covariance matrix is a mathematical way to quantify the shared evolutionary history between two languages (also species, populations, etc.) and to control for phylogenetic non-independence. It provides, for every possible pair of languages in a family, how much of the tree they share. Two closely related varieties will show numbers/proportions close to 1, and far-related ones will show proportions close to 0.

12 More sophisticated options to gauge the phonological similarity of two words can also be pursued through Feature Edit Distance (Kessler, 1995, Heeringa & Nerbonne, 1999, Kondrak, 2000), by which segment replacements are weighted by their distance in articulatory features like +/-consonantal, +/-plosive, +/-bilabial etc.

13 To avoid biases due to non-random absence of datapoints (less basic and therefore more unstable concepts are more likely to be missing), those varieties with over 20% of NA values were discarded.

14 Contact here refers mostly to linguistic influence from non-Romance varieties because of the difficulty to identify contact between closely related varieties. 4 was hence awarded to locations in the immediate vicinity of non-Romance continental contiguous territory (i.e. Germanic or Slavic) in a nation state where a non-Romance language is predominant, and where Romance is historically retreating (i.e. Switzerland, Slovenia, Croatia). 3 was awarded to locations in the immediate vicinity of a location with a score 4, and to locations in the immediate vicinity of non-Romance continental contiguous territory but in a nation state where Romance is predominant (i.e. Italy). Scores 4 and 3, thus, occur only in the Northern and North-Eastern Alpine periphery. 2 was awarded to locations in the immediate vicinity of locations with a score 3, and to locations spoken in the immediate vicinity of areas where Greek is spoken (i.e. in Calabria and Apulia), as well as to the Northern Italian linguistic exclaves in the South. 1 was awarded to locations in the immediate vicinity of locations with a score 2, and to locations in the immediate vicinity of Serbocroatian and Albanian-speaking linguistic exclaves inside southern Italy. 0 was awarded to all other locations far from any non-Romance interference.
